# High-resolution structural study on pyri­din-3-yl ebselen and its *N*-methyl­ated tosyl­ate and iodide derivatives

**DOI:** 10.1107/S2053229623000062

**Published:** 2023-02-17

**Authors:** Ruyi Xu, Thomas Fellowes, Jonathan M. White

**Affiliations:** a School of Chemistry and BIO-21 Institute, University of Melbourne, Parkville, VIC 3010, Melbourne, Australia; University of Sheffield, United Kingdom

**Keywords:** crystal structure, chalcogen bonding, hydrogen bonding, multipole refinement, electron density, ebselen, selenium

## Abstract

The crystal structure of the pyri­dine-substituted benzisoselenazolinone 2-(py­ri­din-3-yl)-2,3-di­hydro-1,2-benzoselenazol-3-one (C_12_H_8_N_2_OSe, **2**), related to the anti­oxidant ebselen [systematic name: 2-phenyl-1,2-benzoselenazol-3(2*H*)-one, **1**], is characterized by strong inter­molecular N⋯Se(—N) chalcogen bonding, where the N⋯Se distance of 2.3831 (6) Å is well within the sum of the van der Waals radii for N and Se (3.34 Å). This strong inter­action results in significant lengthening of the inter­nal N—Se distance, consistent with significant population of the Se—N σ* anti­bonding orbital. Much weaker inter­molecular O⋯Se chalcogen bonding occurs between the amide-like O atom in **2** and the less polarized C—Se bond in this structure. Charge density analysis of **2** using multipole refinement of high-resolution data allowed the electrostatic surface potential for **2** to be mapped, and clearly reveals the σ-hole at the extension of the Se—N bond as an area of positive electrostatic potential. Topological analysis of the electron-density distribution in **2** was carried out within the Quantum Theory of Atoms in Mol­ecules (QTAIM) framework and revealed bond paths and (3,−1) bond critical points (BCPs) for the N⋯Se—N moiety consistent with a closed-shell inter­action; however, the potential energy term is suggestive of electron sharing. Analysis of the electron localization function (ELF) for the strong N⋯Se and the weak O⋯Se chalcogen-bonding inter­actions in the structure of **2** suggest significant electron sharing in the former inter­action, and a largely electrostatic inter­action in the latter. Conversion of **2** to its *N*-methyl­ated derivatives by reaction with methyl iodide [1-methyl-3-(3-oxo-2,3-di­hydro-1,2-benzoselenazol-2-yl)pyri­din-1-ium iodide, C_13_H_11_N_2_OSe^+^·I^−^] and methyl tosyl­ate [1-methyl-3-(3-oxo-2,3-di­hydro-1,2-benzoselenazol-2-yl)pyri­din-1-ium toluene­sulfonate trihydrate, C_13_H_11_N_2_OSe^+^·C_7_H_7_O_3_S^−^·3H_2_O] removes the possibility of N⋯Se chalcogen bonding and instead structures are obtained where the iodide and tosyl­ate counter-ions fulfill the role of chalcogen-bond acceptors, with a strong I^−^⋯Se inter­action in the iodide salt and a weaker *p*-Tol-SO_3_^−^⋯Se inter­action in the tosyl­ate salt.

## Abstract 2

The crystal structure of the pyridine analog of the selenium pharmaceutical ebselen is characterized by one-dimensional N—Se chalcogen-bonded chains where the pyridine N atom is the chalcogen-bond acceptor. Charge density analysis using high-resolution Mo *K*α X-radiation and subsequent multipole refinement reveals a clear region of positive electrostatic potential at the antipode to the Se—N bond of the isoselenazole moiety. In the *N*-methylated iodide and tosylate salts, the iodide/tosylate counter-ions are strongly chalcogen bonded to the Se atom.

## Introduction

**Figure 1:**
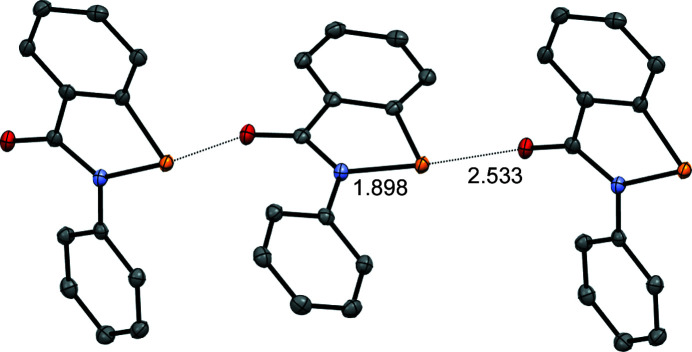
The chalcogen-bonded chains in the crystal structure of ebselen **1**.

The benzisoselenazolinone scaffold of the drug ebselen (**1**) is a potent chalcogen-bond donor, due to the presence of a polarizable Se atom covalently bonded to an electron-withdrawing amide/aniline N atom. The propensity for this system to form chalcogen bonds has been studied within our group and by others, with a view to exploiting it in the context of medicinal chemistry (Thomas *et al.*, 2015 ▸; Fellowes & White, 2019 ▸; Fellowes *et al.*, 2020 ▸, 2022 ▸). However, the concept of chalcogen bonding has also received much attention concerning applications in materials chemistry (Eckstein *et al.*, 2021 ▸). In addition to the chalcogen-bond donor, ebselen also contains an amide carbonyl group which nicely fulfils the role of chalcogen-bond acceptor in directing the crystal packing of this mol­ecule, forming one-dimensional polymers in both polymorphic modifications (Fig. 1 ▸) (Thomas *et al.*, 2015 ▸), held together by Se⋯O=C chalcogen-bond inter­actions, with the Se⋯O=C distances of 2.522 (1) and 2.533 (1) Å being well within the sum of the van der Waals radii for Se and O (3.41 Å) (Bondi, 1964 ▸). The role of chalcogen-bonding inter­actions in the binding of ebselen to the main protease M^pro^ of SARS-CoV-2 has been demonstrated recently (Menendez *et al.*, 2020 ▸; Fellowes & White, 2022 ▸). 
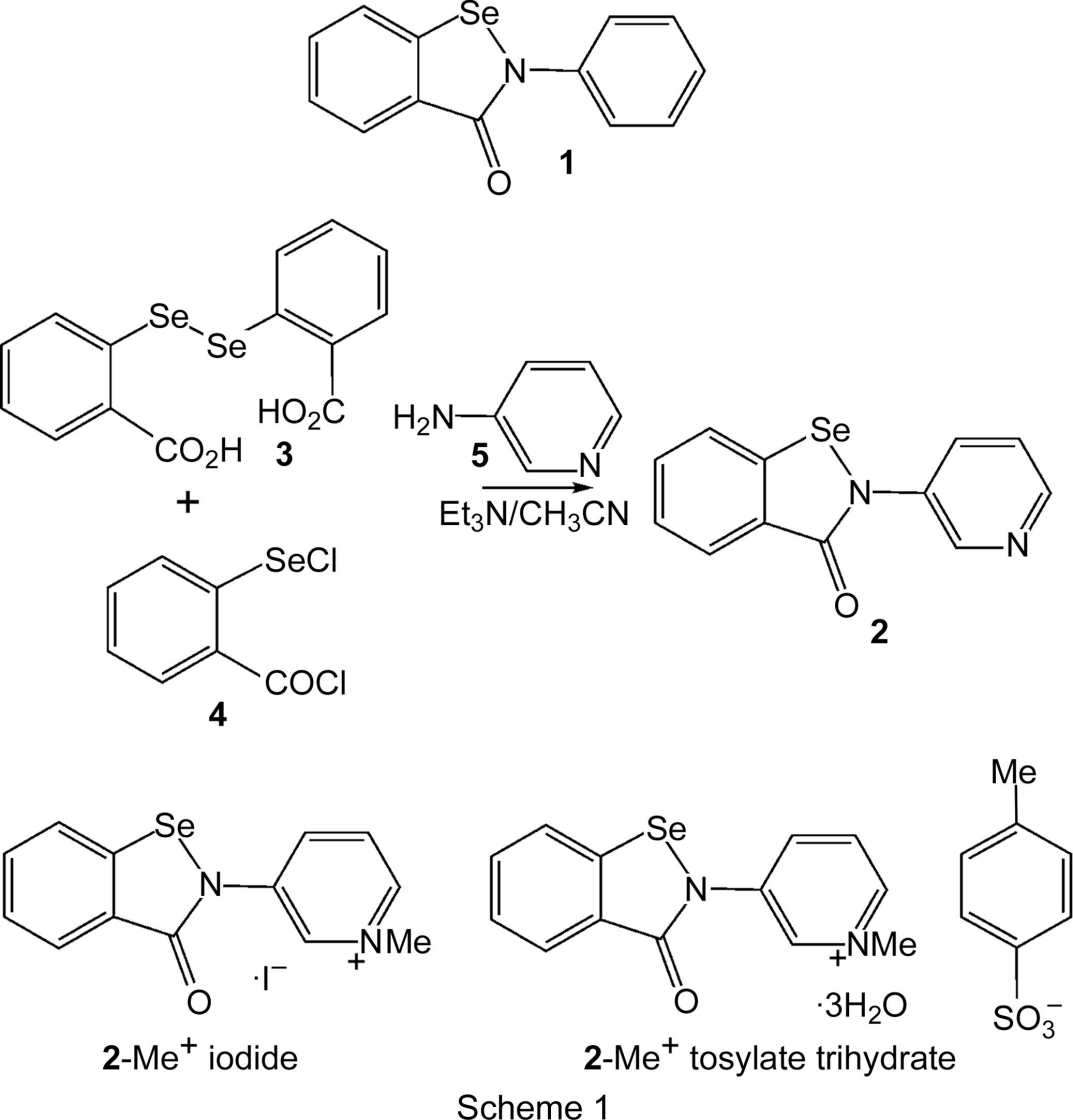


Given the strength of benzisoselenazolinone-based chalcogen-bond donors, we were inter­ested in adapting the system to form other supra­molecular architectures. We envisaged that the pyri­din-3-yl-substituted benzisoselenazolone 2-(pyri­din-3-yl)-2,3-di­hydro-1,2-benzoselenazol-3-one (**2**) might form an alternative one-dimensional chalcogen-bonded polymer, with the pyri­dine N atom fulfilling the role of chalcogen-bond acceptor. Pyridine-substituted ebselen derivative **2** was syn­thesized by reaction of the diselenide 2,2′-(diselane-1,2-di­yl)di­benzoic acid (**3**) with excess thionyl chloride to give the electrophilic inter­mediate 2-(chloro­selan­yl)benzoyl chloride (**4**). The electrophile **4** was then treated with pyri­din-3-amine (**5**) to assemble the benzisoselenazolinone ring (Scheme 1). Crystallization of the crude product from hot di­methyl­formamide (DMF) afforded light-brown plate-like single crystals of **2**.

## Experimental

NMR spectra were recorded on a Varian 400 MHz spectrometer (see supporting information). Chemical shifts are reported in ppm relative to tetra­methyl­silane (TMS), referenced to the residual solvent signal. The integrals are in accordance with assignments and coupling constants are given in Hz. All reported ^13^C spectra are proton decoupled. Multiplicity is indicated as follows: *s* = singlet, *brs* = broad singlet, *d* = doublet, *m* = multiplet and *dd* = doublet of doublets.

### Synthesis and crystallization

#### Preparation of benzisoselenazolinone 2

Diselenide **3** (1.2 g, 2.99 mmol) was refluxed in thionyl chloride (10 ml) for 30 min, after which the solid had dissolved. The excess thionyl chloride was removed by distillation and the residue triturated with dry hexane (20 ml). Removal of the hexane under reduced pressure gave the electrophilic reagent **4** as a pale-yellow solid which was used without further purification or characterization. The electrophilic reagent **4** was then added to a solution of pyri­din-3-amine (**4**; 0.56 g, 2.99 mmol) in aceto­nitrile (10 ml) and anhydrous tri­ethyl­amine (1 ml). The mixture was stirred at room temperature for 2 h, the solvent removed under reduced pressure and the residue crystallized from hot DMF giving com­pound **2** as off-white plates (m.p. 272–274 °C; yield 0.76 g, 90%). ^1^H NMR (*d*_6_-DMSO): δ 8.85 (1H, *brs*), 8.43 (1H, *d*, *J* = 4.7 Hz), 8.08 (1H, *d*, *J* = 8.1 Hz), 8.04 (1H, *ddd*, *J* = 8.3, 7.2, 1.5 Hz), 7.90 (1H, *dd*, *J* = 7.7, 1.4 Hz), 7.68 (1H, *ddd*, *J* = 8.2, 2.7, 1.4), 7.45–7.49 (1H, *m*). ^13^C NMR (*d*_6_-DMSO): δ 166.16, 146.95, 145.97, 139.58, 137.27, 133.12, 132.44, 128.60, 128.45, 126.94, 126.55, 124.59.

#### Preparation of 2-Me^+^ iodide and 2-Me^+^ tosyl­ate

Com­­pound **2** (200 mg) in DMF (5 ml) was heated in the presence of excess methyl iodide (5 equiv.) or methyl tosyl­ate (1.1 equiv.), respectively, giving qu­anti­tative con­version to **2**-Me^+^ iodide (m.p. 268–275 °C, decomposition) and **2**-Me^+^ tosyl­ate (m.p. 212–214 °C). ^1^H NMR for **2**-Me^+^ iodide (*d*_6_-DMSO): δ 9.47 (1H, *dd*, *J* = 1.8, 1.8 Hz), 8.74 (1H, *d*, *J* = 5.9 Hz), 8.71 (1H, *dd*, *J* = 7.6, 0.84 Hz), 8.30 (1H, *d*, *J* = 8.3), 8.11 (1H, *dd*, *J* = 7.7, 1.4), 7.93 (1H, *dd*, *J* = 7.7, 1.4 Hz), 7.7 (1H, *m*), 7.5 (1H, *ddd*, *J* = 8.0, 7.2, 1.0 Hz), 4.37 (3H, *s*). ^1^H NMR for **2**-Me^+^ tosyl­ate (*d*_6_-DMSO): δ 9.52 (1H, *dd*, *J* = 1.9, 1.9 Hz), 8.8 (1H, *d*, *J* = 5.9 Hz), 8.77 (1H, *dd*, *J* = 8.4, 2.3 Hz), 8.05–8.15 (3H, *m*), 7.93 (1H, *dd*, *J* = 7.8, 1.4 Hz), 7.71 (1H, *ddd*, *J* = 8.3, 7.1, 1.5 Hz), 7.51 (1H, *dd*, *J* = 7.5,, 1.0 Hz), 7.10 (2H, *d*, *J* = 7.8 Hz), 4.38 (3H, *s*).

### Refinement

Crystal data, data collection and structure refinement details are summarized in Table 1 ▸. The H atoms were located in difference Fourier maps but were introduced in calculated positions and treated as riding on their parent atoms (C atoms). The H atoms of the water mol­ecules were located in difference Fourier maps and refined isotropically.

**Figure 2:**
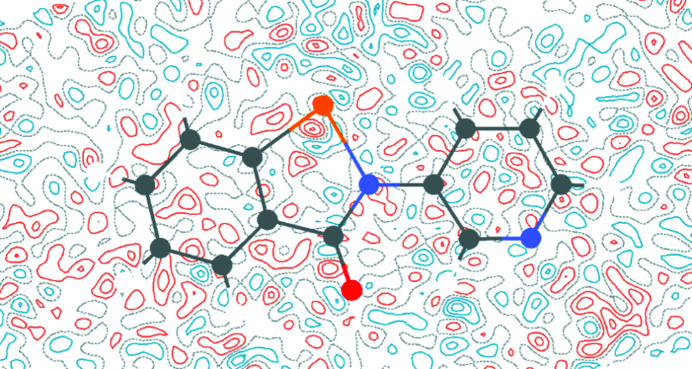
Difference electron residuals from the multipole refinement of com­pound **2** with 0.05 e Å^−3^ contours. Red contours are positive, blue are negative and dashed grey are zero.

Refinements for charge-density analysis of **2** were per­formed against *F* ^2^, up to a maximum reciprocal resolution of 0.95 Å^−1^ for a total of 7174 independent reflections using the *MoPro* software (Guillot *et al.*, 2001 ▸). Beamstop-affected reflections were identified and excluded at the data reduction stage, and disagreable frames were removed. The independent atom model (IAM) structure was first refined using *NoSpherA2* in *OLEX2* (Kleemiss *et al.*, 2021 ▸). This procedure generates aspherical scattering factors for the atoms in the crystal based on a density functional theory (DFT) calculation. The PBE0/def2TZVP level was used for this calculation, and *R*1/*wR*2 values of 0.0281/0.0471 were obtained after con­ver­gence of the wavefunction calculation and crystallographic refinement. This model was used as a starting point for the multipole refinement in the *MoPro Suite* (Guillot *et al.*, 2001 ▸; Jelsch *et al.*, 2005 ▸). As the atomic anisotropic displacement parameters (ADPs) had been adequately determined by refinement using calculated scattering factors, charge density parameters were refined from the beginning, without an initial high-order refinement as is usual in charge density investigations. Statistical weights were used throughout the multipole refinement, and 2% of all reflections were marked as free. Multipole parameters were initialized from the ELMAM2 database for all atoms except for selenium, for which parameters were not available. The multipole expansion was limited to a 32-pole level for the Se atom and to an octupole level for the other heavy atoms. H atoms were modelled at the quadrupole level. Default Slater-type functions were used for all atoms. Charge density symmetry constraints were applied, and kappas were constrained to be equal for chemically equivalent atoms. C—H bonds were constrained to neutron distances and idealized geometries, but *U*_iso_ values were refined freely. Initially, an overall scale factor was refined, and this was included in all subsequent refinements. Valence and multipole population parameters were then refined, followed by their respective kappas, and this cycle was repeated. When this had con­verged (shift/<0.001), *xyz* and *U^ij^* were refined. This procedure was repeated to con­vergence. All heavy atoms were then refined anharmonically (maximum order 3) until con­vergence, and the Gram–Charlier coefficients of each atom were com­pared with their estimated uncertainty. If no coefficient exceeded 3σ, the atom was removed from the anharmonic refinement. Atoms Se1, O1, N2, C2, C3, C5, C7, C8, C9, C10, C11 and C12 displayed appreciable anharmonic motion, and were thus refined as such. An isotropic extinction parameter was introduced, which substanti­ally reduced residual electron density around the Se atom. Kappa constraints were lifted gradually, followed by multipole symmetry constraints, then all parameters were refined together initially with heavy damping, which was reduced to zero in the final cycles. The final *R*1/*wR*2 values were 0.016/0.023 and the goodness-of-fit (GoF) was 1.07. *R*_free_ remained com­parable to *R*1 throughout the refinement, so we do not believe the model suffers from overfitting. The total number of refined parameters in the final cycle was 496, to give a data/parameter ratio of 14.4. The es­tim­ated average error in the electron density was 0.0970 e Å^−3^, with a maximum and minimum residual density of 0.43/−0.41 e Å^−3^, which was randomly distributed through the asymmetric unit (Fig. 2 ▸).

## Results and discussion

Pyridine-substituted benzisoselenazolinone derivative **2** was syn­thesized by reaction of diselenide **3** (Scheme 1) with thionyl chloride giving the electrophilic selenium reagent **4** which was immediately coupled with pyri­din-3-amine (**5**). Crystallization of the crude product from hot DMF afforded light-brown plate-like single crystals of **2** suitable for X-ray analysis. Heating com­pound **2** with methyl iodide or methyl tosyl­ate in DMF gave **2**-Me^+^ iodide and **2**-Me^+^ tosyl­ate, res­pectively. Crystals for all samples were obtained from DMF.

### Structure analysis for 2

**Figure 3:**
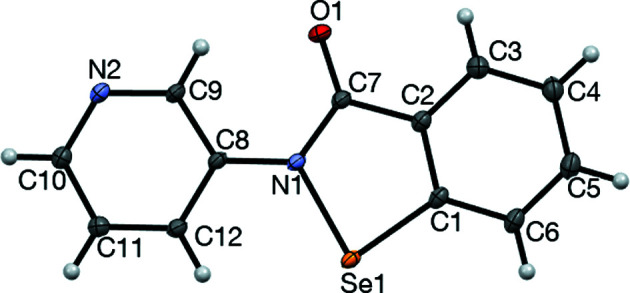
The mol­ecular structure of com­pound **2**, showing 50% probability displacement ellipsoids.

**Figure 4:**
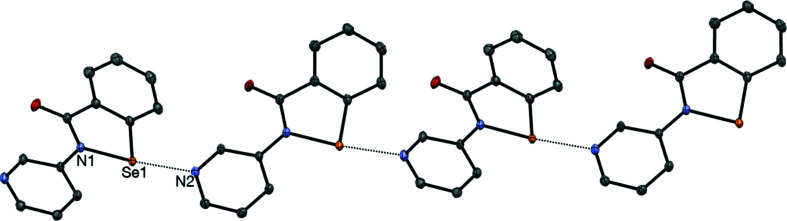
The chalcogen-bonded chains of com­pound **2** propagating along the *ac* diagonal.

**Figure 5:**
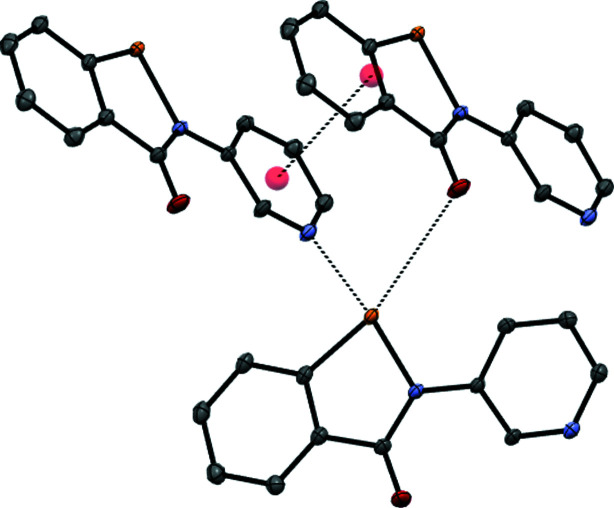
The N⋯Se and O⋯Se chalcogen-bonding inter­actions and π–π stacking inter­actions in the structure of com­pound **2**.

**Figure 6:**
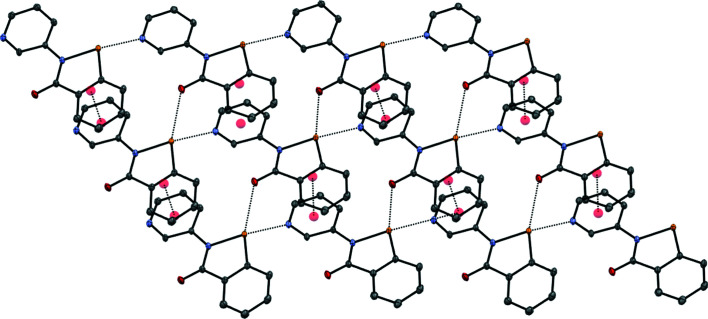
Partial packing diagram of com­pound **2**, showing the three-dimensional network built up of chalcogen-bonding inter­actions and π–π stacking inter­actions, viewed parallel to the (010) plane.

The displacement ellipsoid plot for **2** is presented in Fig. 3 ▸, while selected geometrical parameters are given in Table 2 ▸. The mol­ecular structure is essentially planar, with an r.m.s. deviation of 0.0357 Å for the non-H atoms. The conformation about the N1—C8 bond sees the pyri­dine N atom (N2) on the opposite side to the Se atom (Se1); this conformation may be preferred due to a favourable electrostatic contact between the polarized H atom attached to C9 and amide atom O1, or alternatively this conformation is a consequence of the crystal packing (as discussed below), or both. The structure is characterized by the presence of a strong inter­molecular chalcogen-bonding inter­action involving the polarized Se1—N1 bond [N2^i^⋯Se1 = 2.3831 (6) Å and N2^i^⋯Se1—N1 = 177.44 (2)°; symmetry code: (i) *x* − 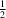 , −*y* + 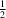 , *z* + 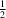 ] which propogates along the *ac* diagonal (Fig. 4 ▸). This strong chalcogen bond combines with a weaker inter­molecular chalcogen-bonding inter­action involving the less polarized Se1—C1 bond with the amide carbonyl O atom [O1^ii^⋯Se1 = 3.3347 (7) Å and O1^ii^⋯Se1—C1 = 165.77 (2)°; symmetry code: (ii) *x* + 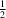 , −*y* + 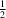 , *z* + 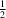 ], and a π-stacking inter­action between the pyri­dine ring [related by the symmetry code (*x* − 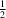 , −*y* + 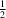 , *z* + 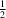 )] and the benzisoselenazolinone ring system [related by the symmetry code (*x* + 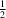 , −*y* + 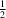 , *z* + 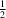 )], having a centroid–centroid distance of 3.412 Å (Fig. 5 ▸). These three inter­molecular inter­actions, while not mutually orthogonal, do result in a three-dimensional supra­molecular network (Fig. 6 ▸). It is worth making a com­parison of the structure of **2**, which contains N⋯Se1(—N1) chains in the crystal, with the parent ebselen (**1**) (Thomas *et al.*, 2015 ▸), which is characterized by C=O⋯Se1—N1 chalcogen-bonded chains. The C=O⋯Se1 inter­action in **1** is characterized by an Se⋯O distance of 2.533 (1) Å (polymorph 2), which represents a contraction of 0.87 Å com­pared to the van der Waals radii for Se and O. In com­parison, the N2⋯Se1(—N1) distance of 2.3831 (6) Å in **2** is contracted by 1.06 Å from the sum of the van der Waals radii for Se and N of 3.45 Å (Bondi, 1964 ▸), suggesting that the N⋯Se(—N) chalcogen-bonding inter­action in **2** is significantly stronger than the O⋯Se(—N) inter­action in **1**. This result is consistent with the pyri­dine N atom in **2** being a significantly stronger chalcogen-bond acceptor than the amide O atom in **1** and agrees with previous results from cocrystal derivatives of **1** (Fellowes *et al.*, 2019 ▸). In addition to dispersion forces, the chalcogen bond has both an electrostatic component (attraction between the positively charged σ-hole on the selenium and the electron-rich chalcogen-bond acceptor) and an orbital inter­action component [in which the electron-rich chalcogen-bond acceptor (highest occupied molecular orbital, HOMO) donates electron density into the low-lying Se—N σ* anti­bonding orbital (lowest unoccupied molecular orbital, LUMO) on the Se atom] (Pascoe *et al.*, 2017 ▸; Kolář & Hobza, 2016 ▸); this latter inter­action results in weakening and lengthening of the inter­nal Se1—N bond distance. Consistent with the apparently stronger N⋯Se inter­action in **2** *versus* the O⋯Se inter­action in **1** is the significant lengthening of the Se1—N1 distance [1.9788 (5) Å] for **2** com­pared to that [1.905 (1) Å] for **1**, suggesting a sig­nificantly increased population of the Se—N σ* anti­bonding orbital in **2**.

### Structure analysis for 2-Me^+^ iodide

**Figure 7:**
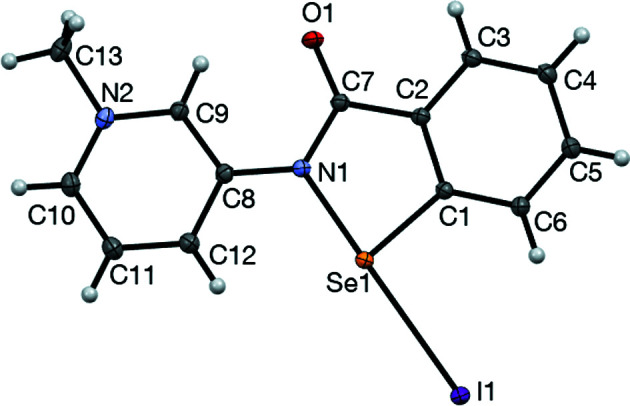
The mol­ecular structure of **2**-Me^+^ iodide, showing 50% probability displacement ellipsoids.

**Figure 8:**
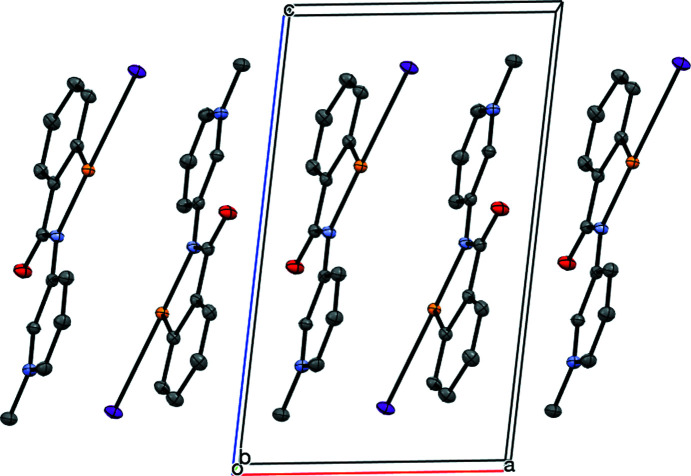
The π–π stacking inter­actions in the structure of **2**-Me^+^ iodide.

The displacement ellipsoid plot for **2**-Me^+^ iodide is pre­sented in Fig. 7 ▸. The structure is essentially planar, with an r.m.s. deviation of 0.038 Å for the non-H atoms of the cation. The iodide counter-ion, which is strongly associated with the cation, lies close to this plane [deviation 0.131 (1) Å]. The nature of the inter­action of the iodide anion with the cation is by an I^−^⋯Se chalcogen-bonding inter­action [I1⋯Se1 = 2.9882 (1) Å and I1⋯Se1—N1 = 178.85 (2)°], which is per­fectly aligned with the anti­pode of the polarized Se1—N1 bond, is well within the sum of the van der Waals radii for I and Se (3.88 Å) and is approaching the bond distance for a formal Se—I covalent bond; the Se—I distance in mesityl selenium iodide is 2.536 (1) Å (Jeske *et al.*, 2002 ▸) and in 2,4,6-tri-*tert*-butyl­phenyl­selenium iodide is 2.529 Å (du Mont *et al.*, 1987 ▸). The strength of this chalcogen bond is not only apparent from the short I^−^⋯Se contact, but also from the significant lengthening of the inter­nal Se—N1 bond distance, which is 2.0053 (6) Å com­pared to 1.905 Å in the parent mol­ecule **1**. Perhaps the I^−^⋯Se1—N1 moiety is best described as a 3-centre–4-electron bond. The crystal packing of **2**-Me^+^ iodide is dominated by strong π–π stacking inter­actions along the *a* axis between mol­ecules of the complex, with each planar mol­ecule sandwiched between two parallel mol­ecules related by the symmetry codes (−*x* + 1, −*y* + 1, −*z* + 1), with an inter­planar spacing of 3.4146 (8) Å, and (−*x* + 2, −*y* + 1, −*z* + 1), with an inter­planar spacing of 3.295 (1) Å (Fig. 8 ▸). Selected geometrical parameters are given in Table 3 ▸.

### Structure analysis for 2-Me^+^ tosyl­ate

**Figure 9:**
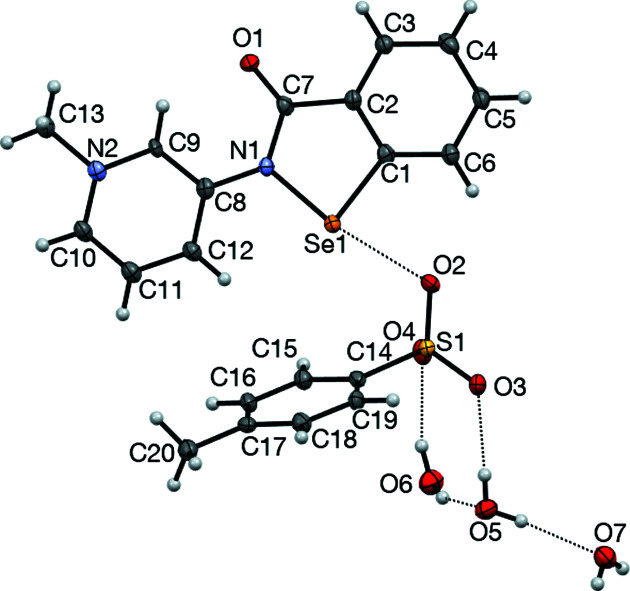
The mol­ecular structure of **2**-Me^+^ tosyl­ate trihydrate, showing 50% probability displacement ellipsoids.

**Figure 10:**
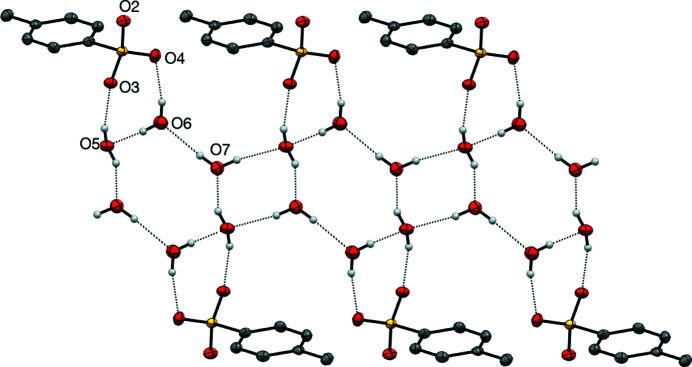
The hydrogen-bonding inter­actions in the structure of **2**-Me^+^ tosyl­ate trihydrate. The undulating chain extends along the *a* axis.

**Figure 11:**
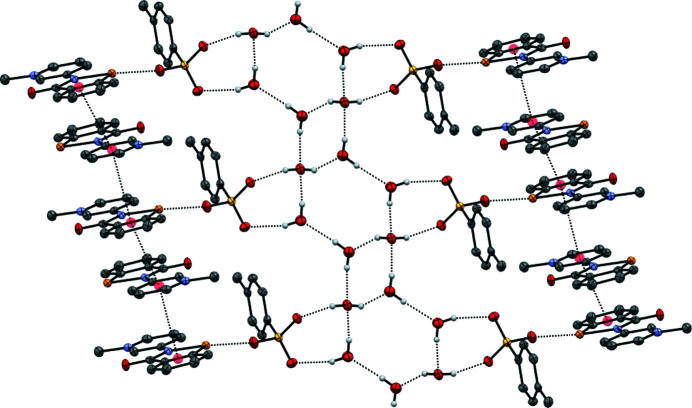
Hydrogen bonding, chalcogen bonding and π–π stacking in the structure of **2**-Me^+^ tosyl­ate trihydrate.

The **2**-Me^+^ tosyl­ate derivative, represented by the displacement ellipsoid plot in Fig. 9 ▸, crystallizes as a trihydrate, which presumably forms as it satisfies the coordination requirements of the tosyl­ate anion, with its three O atoms participating in a number of inter­actions, including a chalcogen-bonding inter­action with the Se atom, in addition to a number of hydrogen-bonding inter­actions involving the three water mol­ecules. The water mol­ecules form an undulating hydrogen-bonded tape parallel to the *a* axis, consisting of alternating six-membered rings fused to four-membered rings, referred to as the T4(2)6(2) motif (Golz & Strohmann, 2015 ▸; Custelcean *et al.*, 2000 ▸). Each six-membered ring provides four hydrogen bonds to two tosyl­ate anions related by inversion (Fig. 10 ▸). The remaining tosyl­ate O atom (O2) forms a chalcogen bond to the Se atom of the cation [O2⋯Se1 = 2.553 (2) Å and O2⋯Se—N1 = 170.57 (10)°]; the planar cations are approproximately orthogonal to the propagating direction of the water tape and allows for inter­digitation from a neighbouring tape by π–π stacking of the benzisoselenazolinone moieties. Each cation is sandwiched between two parallel cations, with inter­planar spacings of 3.383 (7) [at (−*x* + 2, −*y* + 1, −*z* + 1)] and 3.405 (7) Å [at (−*x* + 1, −*y* + 1, −*z* + 1)] (Fig. 11 ▸), resulting in a two-dimensional network parallel to the (0 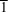 1) plane. Despite the chalcogen-bond inter­action in **2**-Me^+^ tosyl­ate involving a negatively charged tosyl­ate O atom (quenched to a certain extent by the numerous hydrogen-bonding inter­actions), the O2⋯Se1 distance of 2.553 (2) Å (a contraction of 0.857 Å from the sum of the van der Waals radii of 3.41 Å) is clearly weaker than that in the neutral derivative involving the pyri­dine N atom [N2^i^⋯Se1 = 2.3831 (6) Å, a contraction of 1.006 Å from the sum of the van der Waals radii for N and Si of 3.45 Å]. Selected geometrical and hydrogen-bond parameters are given in Tables 4 ▸ and 5 ▸, respectively. Consistent with this is the inter­nal Se—N1 bond of 1.926 (2) Å in **2**-Me^+^ tosyl­ate (Table 6 ▸), which while significantly lengthened com­pared to the parent ebselen (1.905 Å), is much less so than in **2** [Se1—N1 = 1.9788 (5) Å], reflecting the greater extent of the n_N_–σ*_Se—N_ orbital inter­action com­pared to the n_O_–σ*_Se—N_ inter­action.

### Charge density analysis for 2

**Figure 12:**
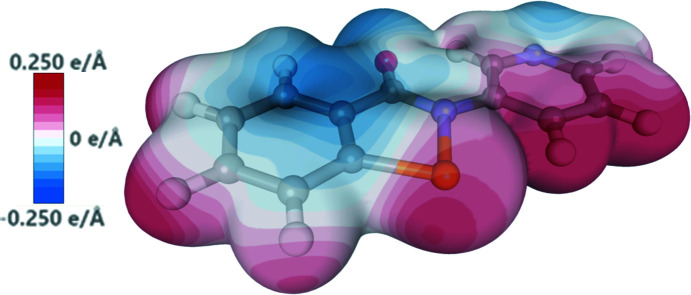
Experimentally determined electrostatic potential for com­pound **2** mapped onto the 0.05 a.u. isosurface.

We used the experimental electron density from the multipole model to explore the electronic features of the chalcogen bond in **2**. Firstly, the electrostatic potential was mapped onto the 0.05 a.u. electron-density isosurface, which revealed a strongly electropositive region along the extension of the Se—N bond, *i.e.* the hole. Also visible were the lone pairs of the O and pyridyl N atom, and electron density above and below the π-system (Fig. 12 ▸).

**Figure 13:**
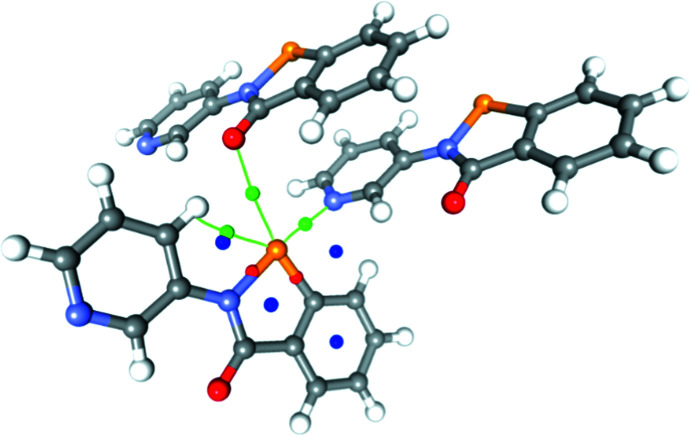
Critical points (CPs) in the vicinity of the Se atom for com­pound **2**. (3,−1) CPs are shown in red (intra­molecular) and green (inter­molecular), and (3,+1) CPs are shown in blue.

The topology of the electron density was also analysed within the QTAIM framework (Bader, 1991 ▸), and bond paths corresponding to the Se⋯N and Se⋯O chalcogen bonds were found, along with associated bond critical points (BCPs; Fig. 13 ▸). The electron density at the BCP for the shorter Se⋯N chalcogen bond of 0.340 e Å^−3^ is significantly larger than that for the Se⋯O chalcogen bond of 0.042 e Å^−3^. The topological parameters associated with these CPs are given in Table 7 ▸. The electron density and Laplacian at the critical point (ρ_CP_ and ∇^2^ρ_CP_ for CP_Se1—N2_) are consistent with a closed-shell inter­action, but we were intrigued by a number of observations which indicate that this may not be the case. Firstly, the endocyclic Se1—N1 bond is lengthened appreciably com­pared to a gas-phase optimized structure [1.9801 (4) *versus* 1.8585 Å; Fellowes & White, 2022 ▸], suggestive of an n_N_–σ* delocalization, leading to partial occupation of the anti­bonding orbital and thus a lengthening of this bond. Secondly, this same bond has similar topological parameters at the CP to those of the chalcogen bond, which may indicate that the Se atom is participating in a 3-centre–4-electron bond between the two N atoms. Notably, the electronic energy density at the critical point *H*_CP_ = *G*_CP_ + *V*_CP_ is less than zero, corresponding to a dominant potential energy term (*V*_CP_), which is strongly indicative of electron sharing (Cremer & Kraka, 1984 ▸; Bone & Bader, 1996 ▸). This can be contrasted with the much weaker Se1—O1 chalcogen bond, where the kinetic energy (*G*_CP_) dominates.

**Figure 14:**
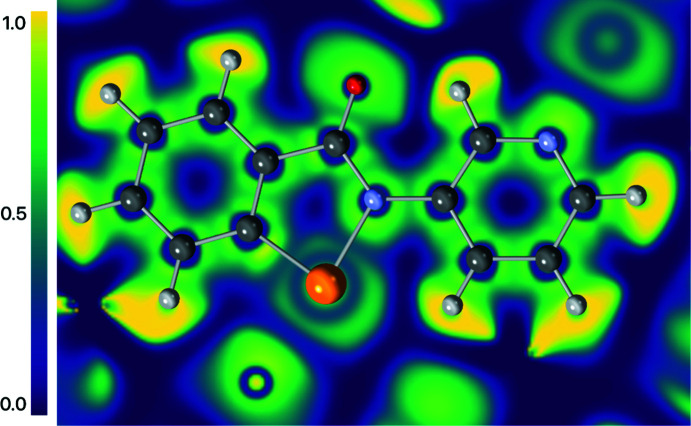
Electron localization function (ELF) in the plane of the ring system for com­pound **2**.

**Figure 15:**
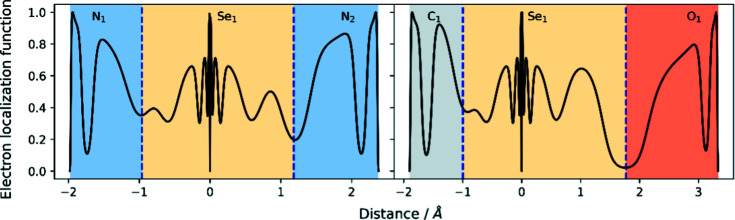
ELF plotted along the N1—Se1—N2 and C1—Se1—O1 bonds for com­pound **2**. BCPs are shown as vertical dashed lines.

The electron localization function (ELF) (Becke & Edgecombe, 1990 ▸) is a measure of the probability of finding an electron of like spin in the vicinity of a fictitious reference electron. It recovers the orbital structure of atoms, while not requiring any knowledge of a wavefunction. An ELF of 1 corresponds to complete localization of an electron pair, a value of 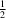 corresponds to a uniform electron gas-like delocalization, while a value of 0 denotes the border between electron pairs. The ELF in the plane of the aromatic system is plotted in Fig. 14 ▸, which shows partial electron localization in the Se1—N1 chalcogen bond, lending further support to the hypothesis that this is not a closed-shell inter­action. The ELF along both the strong N1—Se1—N2 chalcogen bond and the weak C1—Se1—O1 chalcogen bond is plotted in Fig. 15 ▸, clearly showing the difference in ELF at the BCP of these two contrasting cases. In the stronger chalcogen bond, the ELF is approximately 0.2, while in the weak chalcogen bond it is almost zero.

## Conclusions

The crystal structure of the pyri­dine-substituted benzisoselenazolinone **2** is dominated by strong inter­molecular N⋯Se(—N) chalcogen bonding, where the N⋯Se distance of 2.3831 (6) Å is well within the sum of the van der Waals radii for N and Se (3.34 Å). This strong inter­action results in significant lengthening of the inter­nal N—Se distance, con­sistent with a significant orbital inter­action component to the N⋯Se chalcogen bond. Much weaker inter­molecular O⋯Se chalcogen bonding occurs between the amide-like O atom in **2** and the less polarized C—Se bond in this structure. Charge density analysis of 2 using multipole refinement of high-resolution data revealed the presence of a positive electrostatic surface potential at the anti­pode to the Se—N1 bond corresponding to the σ-hole. Topological analysis of the electron-density distribution in 2 within the QTAIM framework revealed bond paths and (3,−1) BCPs for the N⋯Se—N moiety consistent with a closed-shell inter­action. However, the potential energy term suggests a significant contribution from electron sharing. Analysis of the electron localization function (ELF) for the strong N⋯Se and the weak O⋯Se chalcogen-bonding inter­actions in the structure of **2** suggests significant electron sharing in the former inter­action and a largely electrostatic inter­action in the latter. Conversion of **2** to its *N*-methyl­ated derivatives by reaction with methyl iodide and methyl tosyl­ate removes the possibility of N⋯Se inter­molecular chalcogen bonding and instead structures are obtained where the iodide and tosyl­ate counter-ions fulfill the role of chalcogen-bond acceptor, with a strong I^−^⋯Se inter­action in the iodide salt and a weaker *p*-Tol-SO_3_^−^⋯Se inter­action in the tosyl­ate salt.

## Supplementary Material

Crystal structure: contains datablock(s) 2, 2-MeIodide, 2-Metosylate, 2-multipole, global. DOI: 10.1107/S2053229623000062/qz3002sup1.cif

Structure factors: contains datablock(s) 2. DOI: 10.1107/S2053229623000062/qz30022sup2.hkl

Structure factors: contains datablock(s) 2-MeIodide. DOI: 10.1107/S2053229623000062/qz30022-MeIodidesup3.hkl

Structure factors: contains datablock(s) 2-Metosylate. DOI: 10.1107/S2053229623000062/qz30022-Metosylatesup4.hkl

Structure factors: contains datablock(s) 2-multipole. DOI: 10.1107/S2053229623000062/qz30022-multipolesup5.hkl

NMR spectra. DOI: 10.1107/S2053229623000062/qz3002sup6.pdf

CCDC references: 2234063, 2234062, 2234061, 2234060

## Figures and Tables

**Table 1:** Experimental details Experiments were carried out at 100 K using a Rigaku XtaLAB Synergy diffractometer with a Dualflex HyPix detector.

	**2**	**2**-Me^+^ iodide	**2**-Me^+^ tosylate trihydrate	**2**-multipole
Crystal data				
Chemical formula	C_12_H_8_N_2_OSe	C_13_H_11_N_2_OSe^+^·I^−^	C_13_H_11_N_2_OSe^+^·C_7_H_7_O_3_S^−^·3H_2_O	C_12_H_8_N_2_OSe
*M*_r_	275.16	417.10	515.43	275.16
Crystal system, space group	Monoclinic, *P*2_1_/*n*	Triclinic, *P* 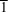	Triclinic, *P* 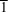	Monoclinic, *P*2_1_/*n*
*a*, *b*, *c* (Å)	6.1087 (1), 14.2241 (2), 12.0630 (2)	7.0926 (1), 8.1329 (1), 11.8376 (1)	6.9412 (3), 12.1279 (4), 13.5994 (3)	6.1074 (1), 14.2227 (3), 12.0621 (2)
α, β, γ (°)	90, 103.594 (1), 90	84.618 (1), 82.243 (1), 77.756 (1)	70.426 (3), 83.774 (3), 83.585 (3)	90, 103.588 (2), 90
*V* (Å^3^)	1018.80 (3)	659.70 (1)	1068.83 (7)	1018.43 (3)
*Z*	4	2	2	4
Radiation type	Mo *K*α	Mo *K*α	Cu *K*α	Mo *K*α
μ (mm^−1^)	3.66	5.18	3.70	3.66
Crystal size (mm)	0.48 × 0.15 × 0.05	0.46 × 0.06 × 0.06	0.21 × 0.03 × 0.03	0.48 × 0.15 × 0.05

Data collection				
Absorption correction	Gaussian (*CrysAlis PRO*; Rigaku OD, 2020 ▸)	Gaussian (*CrysAlis PRO*; Rigaku OD, 2020 ▸)	Multi-scan (*CrysAlis PRO*; Rigaku OD, 2020 ▸)	Gaussian (*CrysAlis PRO*; Rigaku OD, 2020 ▸)
*T*_min_, *T*_max_	0.147, 1.000	0.351, 1.000	0.668, 1.000	0.147, 1.000
No. of measured, independent and observed reflections	98437, 14308, 9929 [*I* > 2σ(*I*)]	90106, 14487, 12038 [*I* > 2σ(*I*)]	15253, 4435, 3777 [*I* > 2σ(*I*)]	97589, 7174, 6298 [*I* ≥ 2u(*I*)]
*R*_int_	0.044	0.055	0.064	0.044
(sin θ/λ)_max_ (Å^−1^)	1.191	1.098	0.632	0.950

Refinement				
*R*[*F* ^2^ > 2σ(*F* ^2^)], *wR*(*F* ^2^), *S*	0.031, 0.072, 1.00	0.025, 0.058, 1.03	0.041, 0.104, 1.06	0.016, 0.023, 1.07
No. of reflections	14308	14487	4435	7174
No. of parameters	145	164	306	496
No. of restraints	0	0	6	22
H-atom treatment	H-atom parameters constrained	H-atom parameters constrained	H atoms treated by a mixture of independent and constrained refinement	All H-atom parameters refined
Δρ_max_, Δρ_min_ (e Å^−3^)	1.25, −0.61	1.24, −1.68	0.89, −0.66	0.43, −0.41

Computer programs: *CrysAlis PRO* (Rigaku OD, 2020 ▸), *SHELXT* (Sheldrick, 2015*a* ▸), *SHELXL2016* (Sheldrick, 2015*b* ▸), *MoPro* (Jelsch *et al.*, 2005 ▸), *Mercury* (Macrae *et al.*, 2020 ▸) and *WinGX* (Farrugia, 2012 ▸).

**Table 2:** Selected geometric parameters (Å, °) for **2** [Chem scheme1]

C1—C2	1.3927 (9)	C8—C9	1.4073 (8)
C1—Se1	1.9037 (6)	C9—N2	1.3388 (8)
C2—C7	1.4715 (8)	C10—N2	1.3354 (9)
C7—O1	1.2349 (8)	N1—Se1	1.9788 (5)
C7—N1	1.3653 (8)	Se1—N2^i^	2.3831 (6)
C8—N1	1.4007 (7)		
			
C2—C1—Se1	112.57 (4)	C7—N1—Se1	115.23 (4)
C6—C1—Se1	128.04 (5)	C8—N1—Se1	120.27 (4)
C1—C2—C7	117.65 (5)	C10—N2—C9	120.38 (5)
N1—C7—C2	110.55 (5)	C1—Se1—N2^i^	90.65 (2)
C7—N1—C8	124.50 (5)	N1—Se1—N2^i^	174.44 (2)

Symmetry code: (i) 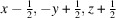 .

**Table 3:** Selected geometric parameters (Å, °) for **2**-Me^+^ iodide[Chem scheme1]

C1—C2	1.3968 (10)	C8—C9	1.4006 (10)
C1—Se1	1.9108 (7)	C9—N2	1.3514 (10)
C2—C7	1.4695 (11)	C10—N2	1.3430 (12)
C7—O1	1.2339 (9)	C13—N2	1.4784 (11)
C7—N1	1.3762 (10)	N1—Se1	2.0053 (6)
C8—N1	1.3907 (10)	Se1—I1	2.9882 (1)
			
C2—C1—Se1	113.17 (5)	C8—N1—Se1	120.73 (5)
C6—C1—Se1	126.93 (6)	C10—N2—C9	122.78 (7)
C1—C2—C7	117.81 (6)	C10—N2—C13	120.40 (7)
N1—C7—C2	110.63 (6)	C9—N2—C13	116.82 (7)
C7—N1—C8	124.27 (6)	C1—Se1—I1	95.85 (2)
C7—N1—Se1	114.94 (5)	N1—Se1—I1	178.85 (2)

**Table 4:** Selected geometric parameters (Å, °) for **2**-Me^+^ tosylate trihydrate[Chem scheme1]

C1—C2	1.398 (4)	C10—N2	1.350 (4)
C1—Se1	1.901 (3)	C13—N2	1.494 (4)
C2—C7	1.471 (4)	C14—S1	1.767 (3)
C7—O1	1.221 (4)	N1—Se1	1.926 (2)
C7—N1	1.388 (4)	O2—S1	1.466 (2)
C8—N1	1.395 (4)	O3—S1	1.459 (2)
C8—C9	1.404 (4)	O4—S1	1.454 (2)
C9—N2	1.352 (4)	O2—Se1	2.553 (2)
			
C6—C1—Se1	126.9 (2)	C8—N1—Se1	119.21 (19)
C2—C1—Se1	112.0 (2)	C10—N2—C9	123.4 (3)
C1—C2—C7	116.9 (3)	C10—N2—C13	119.0 (3)
N1—C7—C2	110.7 (2)	C9—N2—C13	117.6 (2)
C7—N1—C8	125.7 (2)	C1—Se1—N1	85.33 (12)
C7—N1—Se1	115.07 (19)	O2—Se1—N1	170.57 (10)

**Table 5:** Hydrogen-bond geometry (Å, °) for **2**-Me^+^ tosylate trihydrate[Chem scheme1]

*D*—H⋯*A*	*D*—H	H⋯*A*	*D*⋯*A*	*D*—H⋯*A*
C9—H9⋯O1	0.93	2.09	2.734 (4)	125
O5—H5*A*⋯O3	0.82 (1)	1.99 (1)	2.786 (3)	166 (4)
O5—H5*A*⋯S1	0.82 (1)	3.01 (2)	3.698 (2)	144 (3)
O5—H5*B*⋯O7	0.82 (1)	1.99 (4)	2.758 (3)	155 (8)
O6—H6*A*⋯O4	0.82 (1)	2.27 (2)	3.045 (3)	159 (5)
O6—H6*B*⋯O5	0.82 (1)	1.99 (2)	2.782 (4)	162 (7)
O7—H7*A*⋯O6^i^	0.82 (1)	1.97 (1)	2.783 (4)	176 (5)
O7—H7*B*⋯O5^ii^	0.82 (1)	1.97 (1)	2.786 (4)	172 (6)

Symmetry codes: (i) 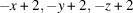 ; (ii) 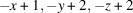 .

**Table 6:** Selected geometric parameters (Å, °) for **2**-multipole

Se1—N1	1.9799 (5)	N2—C10	1.334 (3)
Se1—C1	1.9025 (6)	N2—C9	1.341 (2)
O1—C7	1.243 (2)	C1—C2	1.3914 (18)
N1—C7	1.363 (2)	C2—C7	1.467 (2)
N1—C8	1.3982 (17)	C8—C9	1.412 (2)
			
N1—Se1—C1	84.01 (2)	C10—N2—C9	120.50 (18)
Se1—N1—C7	115.04 (9)	Se1—C1—C2	112.38 (8)
Se1—N1—C8	120.13 (7)	Se1—C1—C6	128.12 (4)
C7—N1—C8	124.83 (11)	N1—C7—C2	110.68 (13)

**Table 7:** Topological parameters at bond critical points (CPs) in the vicinity of the Se atom for **2**

Critical point	Distance	ρ_CP_	∇^2^ρ_CP_	*G*_CP_	V_CP_	ELF
	(Å)	(e A^−3^)	(e A^−5^)	(kJ mol^−1^ Bohr^−3^)	(kJ mol^−1^ Bohr^−3^)	
Se1—N2	2.3807 (9)	0.3402	2.9100	104.7	−130.15	0.196
Se1—O1	3.3328 (7)	0.0420	0.5077	10.81	−7.79	0.022
Se1—N1	1.9801 (4)	0.7782	5.3660	303.37	−460.6	0.351
Se1—C1	1.9028 (3)	0.9873	4.3030	384.34	−651.48	0.374

## References

[bb1] Bader, R. (1991). *Chem. Rev.* **91**, 893–928.

[bb2] Becke, A. D. & Edgecombe, K. E. (1990). *J. Chem. Phys.* **92**, 5397–5403.

[bb3] Bondi, A. (1964). *J. Phys. Chem.* **68**, 441–451.

[bb4] Bone, R. G. A. & Bader, R. F. W. (1996). *J. Phys. Chem.* **100**, 10892–10911.

[bb5] Cremer, D. & Kraka, E. (1984). *Croat. Chem. Acta*, **57**, 1259–1281.

[bb6] Custelcean, R., Afloroaei, C., Vlassa, M. & Polverejan, M. (2000). *Angew. Chem. Int. Ed.* **39**, 3094–3096. 10.1002/1521-3773(20000901)39:17<3094::aid-anie3094>3.0.co;2-p 11028043

[bb7] Eckstein, B. J., Brown, L. C., Noll, B. C., Moghadasnia, M. P., Balaich, G. J. & McGuirk, C. M. (2021). *J. Am. Chem. Soc.* **143**, 20207–20215. 10.1021/jacs.1c08642 34818002

[bb8] Farrugia, L. J. (2012). *J. Appl. Chem.* **45**, 849–854.

[bb9] Fellowes, T., Skene, C. E., Martin, R. F., Lobachevsky, P., Owyong, T. C., Hong, Y. & White, J. M. (2022). *Arkivoc*, pp. S1–S14.

[bb10] Fellowes, T., Van Koeverden, M. P. & White, J. M. (2020). *Cryst­EngComm*, **22**, 4023–4029.

[bb11] Fellowes, T. & White, J. M. (2019). *CrystEngComm*, **21**, 1539–1542.

[bb12] Fellowes, T. & White, J. M. (2022). *J. Mol. Model.* **28**, 66. 10.1007/s00894-021-05023-5 PMC8867462 35201444

[bb13] Golz, C. & Strohmann, C. (2015). *Acta Cryst.* E**71**, 564–566. 10.1107/S2056989015008105 PMC4420053 25995881

[bb14] Guillot, B., Viry, L., Guillot, R., Lecomte, C. & Jelsch, C. (2001). *J. Appl. Cryst.* **34**, 214–223.

[bb15] Jelsch, C., Guillot, B., Lagoutte, A. & Lecomte, C. (2005). *J. Appl. Cryst.* **38**, 38–54.

[bb16] Jeske, J., Jones, P. G., von Salzen, A. M. & Mont, W.-W. (2002). *Acta Cryst.* E**58**, o350–o352.

[bb17] Kleemiss, F., Dolomanov, O. V., Bodensteiner, M., Peyerimhoff, N., Midgley, L., Bourhis, L. J., Genoni, A., Malaspina, L. A., Jayatilaka, D., Spencer, J. L., White, F., Grundkötter-Stock, B., Steinhauer, S., Lentz, D., Puschmann, H. & Grabowsky, S. (2021). *Chem. Sci.* **12**, 1675–1692. 10.1039/d0sc05526c PMC8179328 34163928

[bb18] Kolář, M. H. & Hobza, P. (2016). *Chem. Rev.* **116**, 5155–5187. 10.1021/acs.chemrev.5b00560 26840433

[bb19] Macrae, C. F., Sovago, I., Cottrell, S. J., Galek, P. T. A., McCabe, P., Pidcock, E., Platings, M., Shields, G. P., Stevens, J. S., Towler, M. & Wood, P. A. (2020). *J. Appl. Cryst.* **53**, 226–235. 10.1107/S1600576719014092 PMC6998782 32047413

[bb20] Menendez, C. A., Bylehn, F., De Perez-Lemus, G. R., Alvarado, W. & Pablo, J. J. (2020). *Sci. Adv.* **6**, eabd3045. 10.1126/sciadv.abd0345 PMC7486088 32917717

[bb21] Mont, W. du, Kubiniok, S., Peters, K. & von Schnering, H. (1987). *Angew. Chem. Int. Ed. Engl.* **26**, 780–781.

[bb22] Pascoe, D. J., Ling, K. B. & Cockroft, S. L. (2017). *J. Am. Chem. Soc.* **139**, 15160–15167. 10.1021/jacs.7b08511 28985065

[bb23] Rigaku OD (2020). *CrysAlis PRO*. Rigaku Oxford Diffraction Ltd, Yarnton, Oxfordshire, England.

[bb24] Sheldrick, G. M. (2015*a*). *Acta Cryst.* A**71**, 3–8.

[bb25] Sheldrick, G. M. (2015*b*). *Acta Cryst.* C**71**, 3–8.

[bb26] Thomas, S. P., Satheeshkumar, K., Mugesh, G. & Guru Row, T. (2015). *Chem. Eur. J.* **21**, 6793–6800. 10.1002/chem.201405998 25766307

