# Supplementary figures and images for: High-resolution structural study on pyri­din-3-yl ebselen and its N-methyl­ated tosyl­ate and iodide derivatives
Source: Acta Crystallogr C Struct Chem. 2023 Feb 17;79(Pt 2):43–51. doi: 10.1107/S2053229623000062 (PMC9899513; doi:10.1107/S2053229623000062)

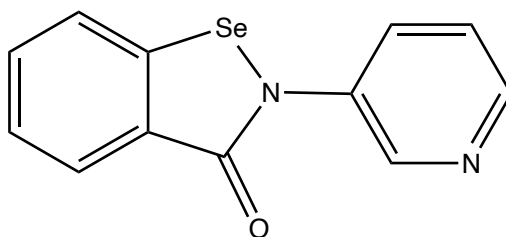

$^1\text{H}$  NMR spectrum (free base)

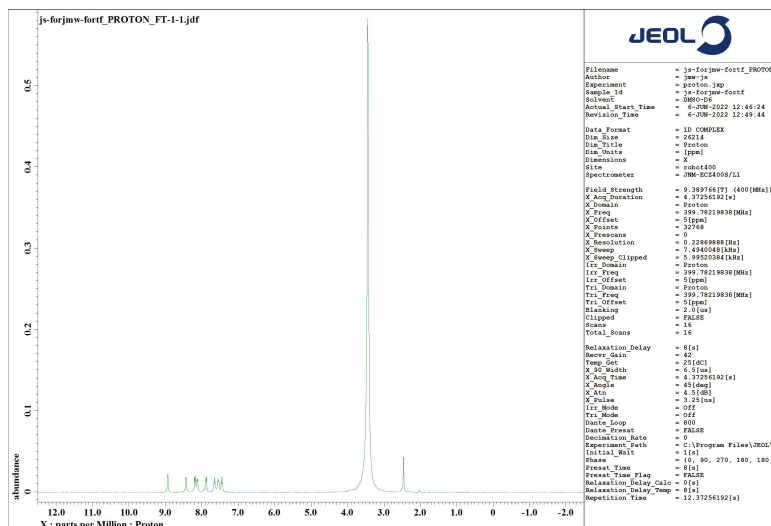

# $^{13}\text{C}$ NMR spectrum (free base)

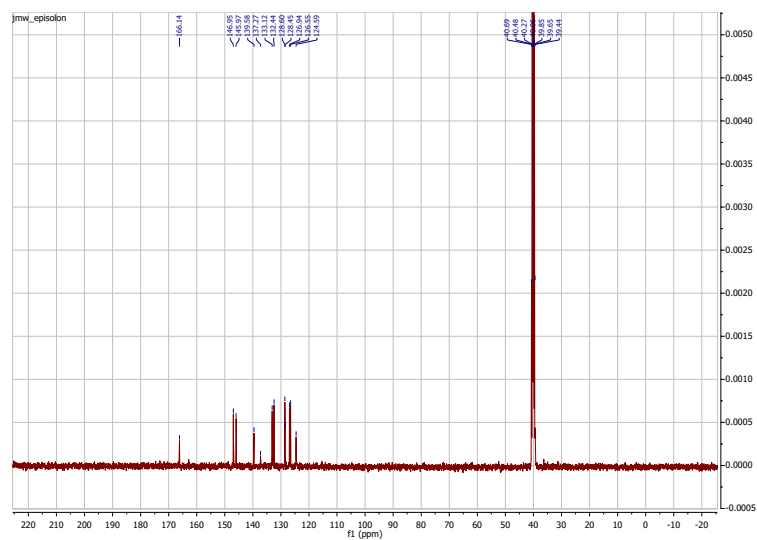

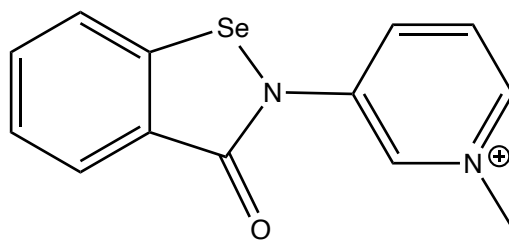

<sup>1</sup>H NMR spectrum (iodide salt)

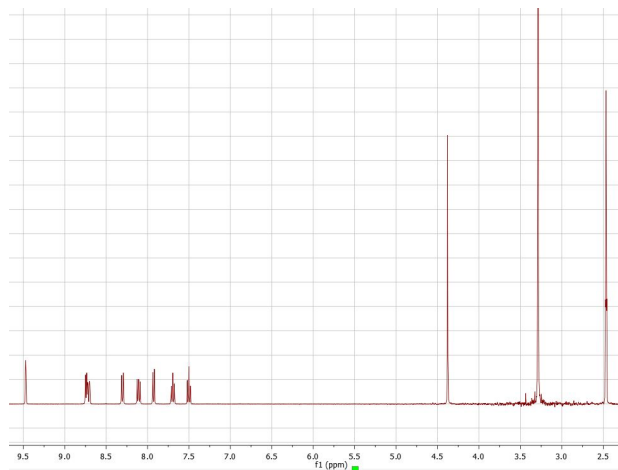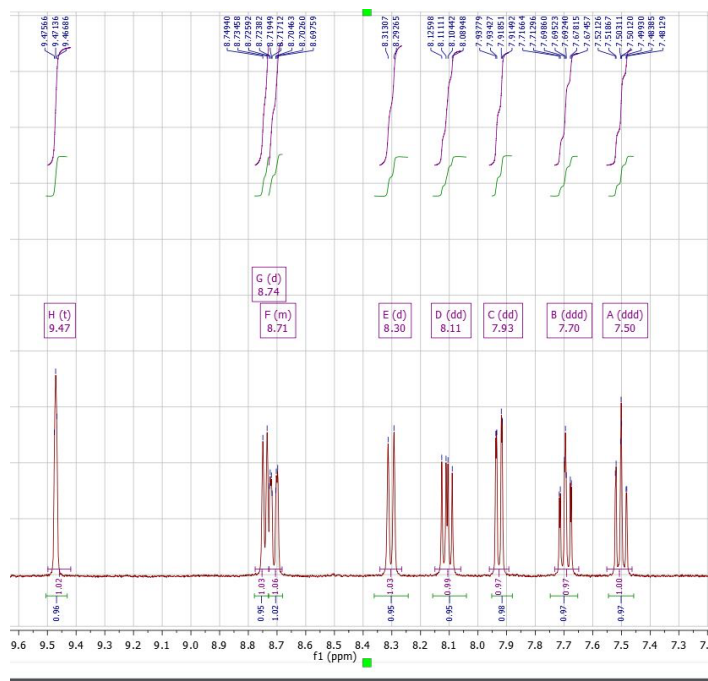

Supplement: Supplementary file 6 [file c-79-00043-sup6.pdf]
